# Utility of Serum Interleukine-6 Level in Empty Nose Syndrome

**DOI:** 10.7150/ijms.83993

**Published:** 2023-05-15

**Authors:** Hsiang-An Hsueh, Ta-Jen Lee, Chi-Che Huang, Po-Huang Chang, Chia-Hsiang Fu

**Affiliations:** 1 Department of Otolaryngology-Head and Neck Surgery, Linkou Chang Gung Memorial Hospital, Taoyuan, Taiwan.; 2 Graduate Institute of Clinical Medical Sciences, College of Medicine, Chang Gung University, Taoyuan, Taiwan.

**Keywords:** interleukin-6 (IL-6), depression, Beck depression inventory-II (BDI-II), biomarker, empty nose syndrome

## Abstract

**Objectives:** Empty nose syndrome (ENS), a complication resulting from surgical procedures on turbinate tissue, is characterized by paradoxical nasal obstruction with wide nasal airways. Patients with ENS often also experience psychiatric symptoms, and psychiatric disorder detection remains dependent on subjective evaluation. Objective biomarkers for mental status assessment in patients with ENS are unestablished. This study aimed to evaluate the role of serum interleukin-6 (IL-6) levels in the mental status of patients with ENS.

**Methods:** Overall, 35 patients with ENS who underwent endonasal submucosal implantation surgery were prospectively included in the study. The Sino-Nasal Outcome Test-25 (SNOT-25), Empty Nose Syndrome 6-item Questionnaire (ENS6Q), Beck Anxiety Inventory (BAI), and Beck Depression Inventory-II (BDI-II) were used to assess the physical and psychiatric symptoms of these patients preoperatively, and 3, 6, and 12 months postoperatively. Serum IL-6 levels were analyzed 1 day before surgery.

**Results:** All subjective assessments significantly improved 3 months after surgery and plateaued at 12 months. Patients with higher serum preoperative IL-6 levels tended to experience more severe depression. Regression analysis showed that a preoperative serum IL-6 level > 1.985 pg/mL was significantly correlated with severe depression status in patients with ENS (odds ratio = 9.76, *p* = 0.020).

**Conclusions:** ENS patients with higher preoperative serum IL-6 levels were more likely to have severe depressive burden. Since more suicidal thoughts or attempts were noted in these patients, timely treatment plan for patients with high levels of serum IL-6 is crucial and may consider psychotherapy after surgical treatment.

## Introduction

The term empty nose syndrome (ENS) was first introduced in 1994, originally to describe certain symptomatology.^1^, ^2^ Diagnosis is made based on clinical findings, although there is poor correlation between clinical findings and patients' symptoms. In addition, ENS does not occur in all patients undergoing turbinate resection.^3^ Most patients have subjective nasal obstruction despite objectively patent nasal cavities on clinical examination; this is termed as “paradoxical obstruction.” Other symptoms include sensation of crusting, dryness, hyposmia, an exaggerated feeling of patency in the nostrils, and suffocation.^2^

Chronic and debilitating conditions also lead to heavy psychiatric burden in those with ENS, causing chronic fatigue, frustration, irritability, anger, anxiety, and depression.^4^, ^5^ The degree and severity of depression in patients with ENS was higher than that in patients with other sinonasal diseases.^6^, ^7^ Lemogne et al. revealed that psychological symptoms in ENS patients could be treated using either antidepressant agents or cognitive behavior therapy^8^, or both, in conjunction with each other. Moreover, the main goal of surgery in ENS treatment is to reconstitute the anatomical structure of the nasal cavity to restore physiological airflow^9^, and our previous investigation showed that endonasal submucosal implantation could significantly improve depression and anxiety symptoms.^4^, ^10^

Approximately 70% of patients with ENS have been shown to experience depression and anxiety.^4^, ^6^, ^11^ The severity of ENS symptoms was found to be directly correlated to psychiatric disease severity.^5^, ^11^, ^12^ However, despite several subjective measurements applied to patients with ENS^4^, ^5^, there is still a lack of reliable objective biomarkers for evaluating their mental status. Our previous investigation found that the preoperative serum high-sensitivity C-reactive protein (hs-CRP) level may be a feasible predictor of surgical outcome regarding depression status in patients with ENS.^13^

Neurotransmitters, which are thought to play a pivotal role in major depressive disorder (MDD), are affected by the cytokines within the brain.^14^ Studies have demonstrated that among pro-inflammatory cytokines, interleukin-6 (IL-6) may play a role in depressive disorders.^15^, ^16^ IL-6 is a pleotropic cytokine produced in response to tissue damage and infections and is promptly synthesized by myeloid cells.^17^ When stress causes neuroinflammation, the permeability of blood brain barrier (BBB) may be potentially increased, allowing pro-inflammatory cytokines, such as IL-6 access to the brain via the highly permeable regions of the BBB.^18^, ^19^ Elevated IL-6 might then cause hypothalamic-pituitary-adrenal axis, which is crucial to deal with stress, dysfunction, alterations in synaptic neurotransmission, and reducing neurotrophic factor.^20^ With several studies suggesting that increased inflammation cytokine levels could cause depression, serum IL-6 might serve as a feasible biomarker for assessing depression status.^21^-^26^ This study aimed to evaluate the association between serum IL-6 levels and psychiatric status in patients with ENS.

## Material and Methods

### Patient enrollment

This prospective study was conducted in accordance with the Declaration of Helsinki and approved by the Institutional Review Board of Chang Gung Memorial Hospital (IRB No. 201902001A3). The diagnosis of ENS was made in patients with typical ENS symptoms, such as paradoxical nasal obstruction, dryness, diminished airflow, or suffocation, who met the following criteria: (1) a history of turbinectomy; (2) endoscopic and/or computed tomography (CT) evidence of turbinate volume reduction; (3) a positive cotton test; (4) Empty Nose Syndrome 6-Item Questionnaire (ENS6Q) scores of **≥** 11^27^; and (5) patients who eventually underwent endonasal submucosal implantation. Patients with the following criteria were excluded in this study: (1) history of any psychiatric disorders or use of antipsychotic medication prior to the first turbinate surgery; (2) congenital craniofacial anomaly or nasal deformity resulting from trauma or rhinoplasty; (3) other sinonasal diseases, such as nasal polyps or rhinosinusitis; and (4) history of sinonasal neoplasm or radiation therapy.

### Surgical intervention and postoperative regimen

The surgical procedure has been described in detail in our previous study.^13^ Briefly, endonasal submucosal implantation was performed with porous high-density polyethylene (Ultra-Thin Sheet Medpor ®, Porex Surgical, Inc., Newnan, GA, USA). All surgical procedures were performed by the same surgeon on all patients included in the study. An incision was made at the nasal floor over the pyriform aperture to create a submucosal pocket. Medpor®, a nonreactive material that allows tissue and vascular ingrowth, was cut into 8 × 25 mm^2^ to 8 × 40 mm^2^ pieces. After filling the submucosal space with small pieces of Medpor, the elevated mucosal flap was repositioned to create an intact augmentation surface. Nasal packing against the implant in the lateral wall was then performed to stabilize the implants. All patients underwent postoperative nasal debridement weekly for the first month. Regular endoscopic follow-up was performed monthly for 6 months and every 2 months thereafter.

### Measurement of associated parameters

Age, sex, smoking status, and allergies were recorded. Other associated factors, such as high body mass index (BMI), smoking, and comorbidities that may affect serum IL-6 levels^20^, were also recorded. The patients' blood samples were collected one day before the operation. Serum IL-6 levels were analyzed via electrochemiluminescent immunoassay (ECLIA) using a Roche Cobas® e 801 Module, with a detection range of 1.5 to 5000 pg/mL.

Sino-Nasal Outcome Test-25 (SNOT-25) and ENS6Q were administered before and after surgery. SNOT-25 aims to assess ENS-specific symptoms and is commonly used for evaluating postoperative outcomes in patients with ENS.^28^ ENS6Q, first developed in 2016, is a validated specific questionnaire for identifying patients suspected of developing ENS.^27^

To analyze psychiatric status, the Beck Depression Inventory-II (BDI-II) and Beck Anxiety Inventory (BAI) were administered. First developed in 1961 and revised in 1996 by Beck, BDI-II was widely used for assessing the severity of depression in adolescents and adults.^29^ BAI, also developed by Beck in 1990, is a 21-item self-report instrument for measuring the severity of anxiety in adolescents and adults.^30^ For the BDI-II, total scores of 0-13, 14-19, 20-28, and 29- 63 indicate normal, mild, moderate, and severe depression, respectively; and for BAI, total scores of 0-7, 8-15, 16-25, and 26-63 indicate normal, mild, moderate, and severe anxiety, respectively.

The SNOT-25, ENS6Q, BDI-II, and BAI were completed by all patients enrolled in the study 1 week before the operation, and all questionnaires were readministered at 3, 6, and 12 months postoperatively.

### Data analysis

Statistical analyses were performed using SPSS Statistics for Windows version 22.0. (Armonk, NY: IBM Corp.). The Wilcoxon signed-rank test was used to compare pre- and postoperative questionnaire results. Data is presented as mean ± standard deviation (SD). The association between IL-6 levels and other potential medical factors was analyzed using simple linear regression analysis. Intergroup comparisons were performed using Fisher's exact test and Mann-Whitney *U* test for categorical and continuous variables, respectively. Linear regression was used to evaluate the association between symptoms and other potential factors. Using the receiver operating characteristic (ROC) curve, the ideal cut-off value of the serum IL-6 level for predicting preoperative psychiatric status was obtained based on Youden's index. Multivariate logistic regression models were constructed using forward selection (*p* = 0.01) and backward elimination (*p* = 0.05) to identify independent patient characteristics associated with preoperative psychiatric status. Odds ratios (OR) and 95% confidence intervals (CI) were also calculated for type I errors. A two-tailed *p* < 0.05 was considered statistically significant.

## Results

### Patient characteristics

From July 2019 to December 2022, forty-nine patients were initially enrolled in this study. Of these, 14 patients who did not complete postoperative assessments were excluded. The final sample comprised of 35 male patients (*n* = 26, 74%). The mean age of the patients was 47.0 ± 12.2 years (range, 24-68 years). Seven of these patients had chronic medical disease diagnosed prior to surgical treatment, which was recorded as a comorbidity (each patient had a different comorbidity: acute hepatitis, psoriasis, hyperlipidemia, hypertension, coronary artery disease, glaucoma, and pertussis, respectively). Demographic data, laboratory parameters, and preoperative subjective measurements are shown in Table 1.

### Subjective assessments before and after surgery

**Figure 1:**
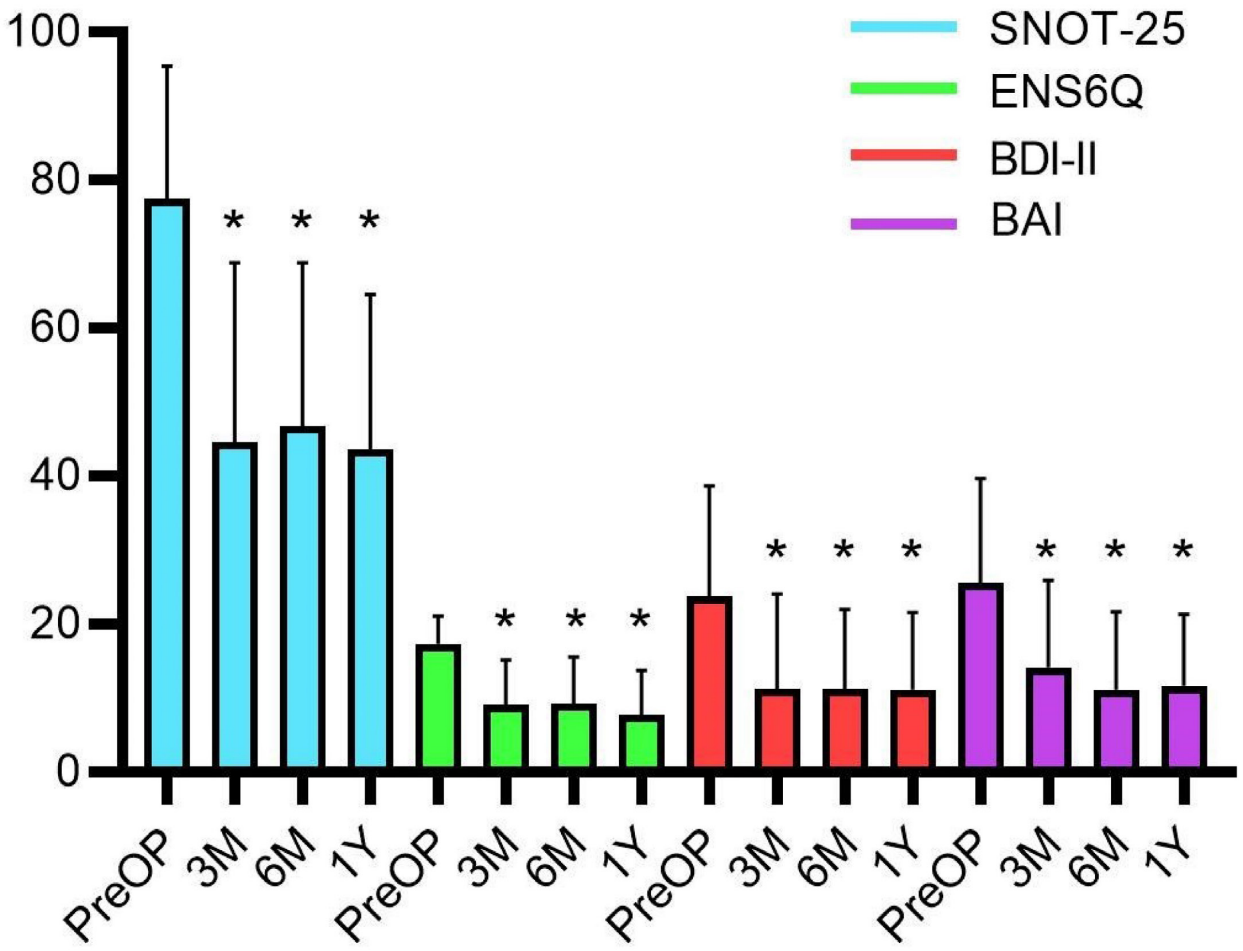
The subjective assessments before and after surgical treatment with Wilcoxon signed-rank test. The postoperative BDI-II, BAI, ENS6Q and SNOT-25 scores significantly improved at the third months and plateaued until one year after surgery. Data were presented as mean ± standard deviation. BAI: Beck anxiety inventory; BDI-II: Beck depression inventory‐II; ENS6Q: Empty Nose Syndrome 6‐Item Questionnaire; M: month; PreOP: preoperative; SNOT-25: Sino‐Nasal Outcome Test‐25; Y: year. * *p*-values < 0.05 compared with preoperative status.

The preoperative mean BDI-II was measured to be 23.7 ± 14.9; and 37.1% of patients were classified under the severe depression group. The preoperative mean BAI score was 25.5 ± 14.1; and almost half of the patients (48.6%) were classified under the severe anxiety group (Table 1). After the operation, all subjective assessments, including the SNOT-25, ENS6Q, BDI-II, and BAI, showed significant improvements (all *p* < 0.0001) in both nasal symptoms and psychiatric assessments. A marked improvement was noted after the third month and persisted until one year after surgery (Figure 1).

### Correlation and ROC curve analysis

**Figure 2:**
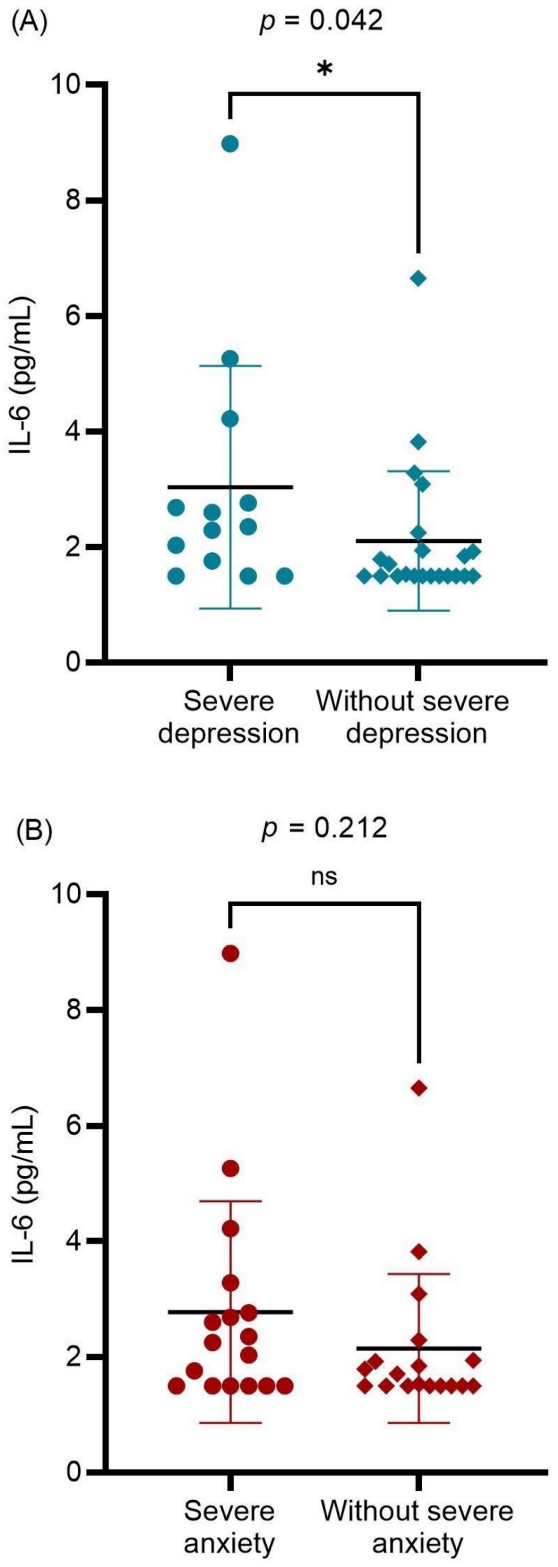
The differences in the preoperative serum IL-6 level between preoperative (A) severe and without severe depression groups; (B) severe anxiety and without severe anxiety groups. Data were presented as mean ± standard deviation and analyzed with Mann-Whitney *U* test. IL-6: interleukin-6. * *p* < 0.05 statistically significant.

The relationship between preoperative serum IL-6 levels and other potentially associated factors were analyzed (Table 2). A significantly positive correlation was found between serum IL-6 levels and preoperative subjective measurements (ENS6Q, SNOT-25, BDI-II, and BAI scores; *p* = 0.034, *p* = 0.021, *p* = 0.022, and *p* = 0.031, respectively). Age was also significantly associated with preoperative serum IL-6 levels. Further evaluation of mental status revealed that patients with ENS with severe depression had higher serum IL-6 levels than others (*p* = 0.042; Figure 2A). In contrast, preoperative severe anxiety status was not associated with IL-6 levels (*p* = 0.212; Figure 2B). The results indicated that ENS patients with higher serum IL-6 levels tended to suffer from severe depression before surgical treatment. There was no significant correlation between the preoperative serum IL-6 levels and postoperative subjective measurements. Furthermore, no significant difference in serum IL-6 levels was detected between the postoperative depression and non-depression groups (*P* = 0.439). Further, among the enrolled patients, only 21 patients received postoperative serum IL-6 level measurements at 12 months after surgery. No significant difference was detected between pre- and postoperative serum IL-6 levels (*p* = 0.594). Nevertheless, we found those in severe depression before surgical treatment (10 patients) showed a decrease in the serum IL-6 level with marginal significance (3.30 pg/mL vs. 1.94 pg/mL, *p* = 0.047). In the correlation analysis, postoperative serum IL-6 level had no marked relation with the subjective measurements (all *p* > 0.05).

**Figure 3:**
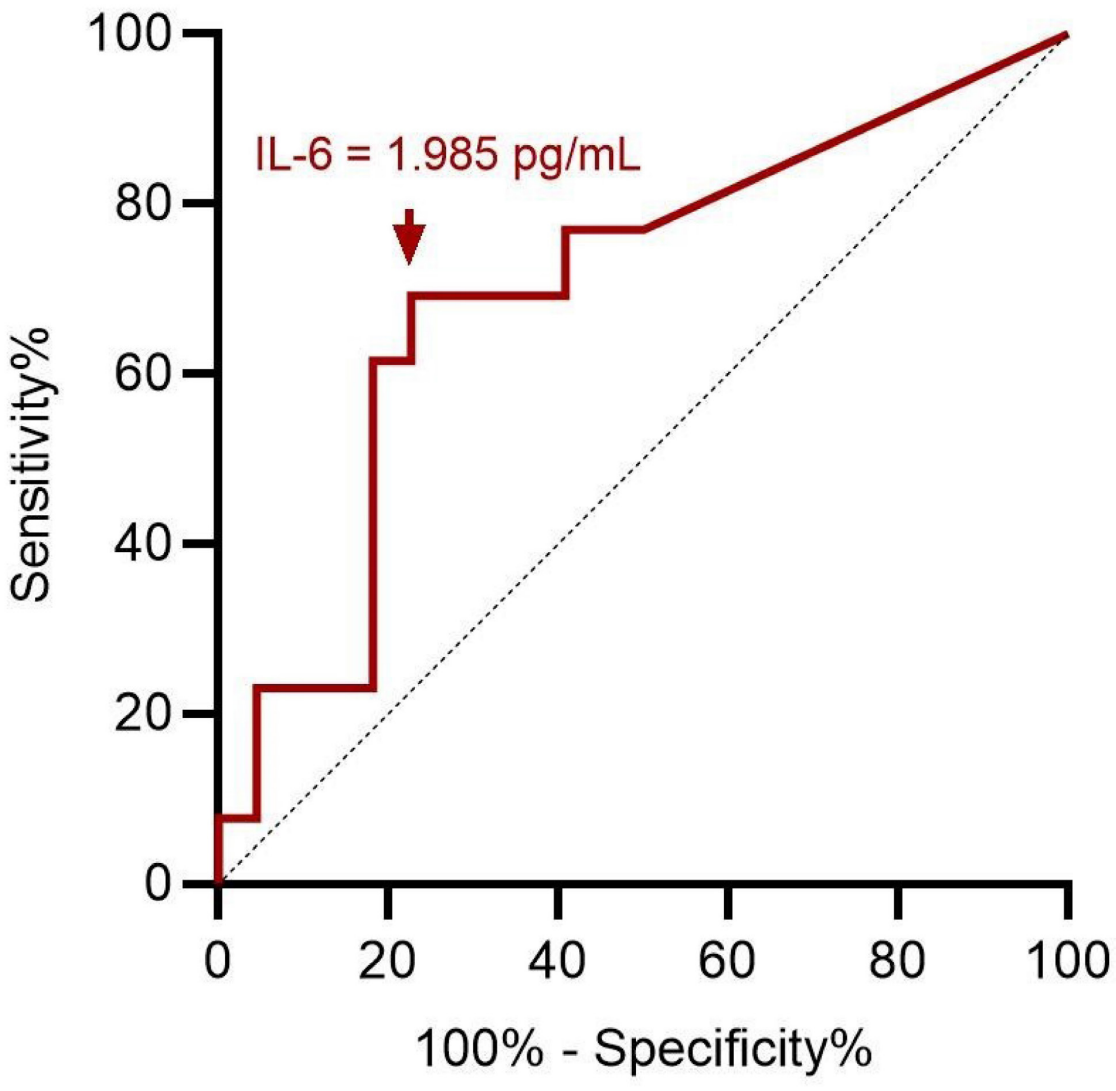
ROC curve analysis for preoperative IL-6 in predicting preoperative severe depression status. A cut-off value of preoperative serum IL-6 value at 1.985 pg/mL has optimal sensitivity and specificity according to Youden index. ROC: receiver operating characteristic; IL-6: interleukin-6.

ROC curve analysis showed an optimal cut-off value of 1.985 pg/mL for preoperative serum IL-6 level to assess preoperative severe depression status (*p* = 0.049; Figure 3). This implies that patients with ENS were more likely to experience severe depression when they had a preoperative IL-6 level > 1.985 pg/mL, with 69.2% sensitivity and 77.3% specificity (Table 3). With ENS, patients were classified into two groups based on the cut-off value of the preoperative serum IL-6 level, and no significant difference in postoperative subjective outcomes was found between the two groups.

### Regression analysis

Univariate logistic regression analysis showed that preoperative severe depression was associated with a higher preoperative serum IL-6 level (> 1.985 pg/mL) (*p* = 0.010). To eliminate the influence of other potentially associated factors, multivariate logistic regression was further applied, which showed a significant correlation (*p* = 0.020). The odds ratio of a higher preoperative serum IL-6 level was measured to be 9.76, indicating that ENS patients with a higher serum IL-6 level would have a nearly 10-fold risk of developing severe depression (Table 4).

## Discussion

It has been demonstrated that patients with ENS have a probability to experience psychiatric disorders such as depression and anxiety.^4^, ^5^ This significant psychiatric disease burden leads to difficulties in many activities of daily living. Mental illness also causes heavy social and economic burden. Health care utilization costs have been reported to be three and a half times higher, while social services costs are three times higher, among patients with depression compared to patients without depression.^31^ The diagnosis of a psychiatric disorder mainly depends on subjective measurements of associated symptoms. Therefore, obtaining a feasible and objective biomarker to assess mental disorders would be more measurable and practical. IL-6 has been reported to be linked to stress-related disorders, such as depression and anxiety.^32^ To the best of our knowledge, this is the first study on the association between serum IL-6 levels and depression status in patients with ENS.

IL-6 is present at low levels in peripheral blood but increases in patients with markers of frailty, during infection, trauma, chronic disease, or other stress.^33^, ^34^ It is a pleiotropic cytokine that affects various biological systems and organs.^23^ IL-6 may increase the activity of indoleamine-2,3-dioxygenase, which is involved in tryptophan metabolism, leading to kynurenine pathway activation and decreased central serotonin availability. Furthermore, as previous studies have revealed, plasma IL-6 levels increase with age in healthy individuals^34^-^36^, our investigation of ENS patients had similar results. Other associated factors, including BMI, smoking, and comorbidities^20^, were not significantly correlated with serum IL-6 levels in our study.

Serum IL-6 levels in different depression subtypes have been investigated and certain subtypes, such as melancholic and atypical depression, have higher peripheral IL-6 levels compared with others.^20^ About the association between IL-6 levels and depression severity, Fan et al. found that serum IL-6 level is positively correlated with Hamilton Depression Scale-17 scores for major depressive disorder (MDD) patients.^37^ In another study of 118 MDD patients, plasma IL-6 levels reflected the severity of MDD, inferring that plasma IL-6 may be another biological marker for the depressive state.^38^ The authors also found the plasma levels of IL-6 were significantly higher in the selective serotonin reuptake inhibitors responders than in the non-responders. Under this stress, the increased serum IL-6 level might be associated with the central serotonin pathway, resulting in depression symptoms. The complex pathophysiology between IL-6 and ENS deserves further investigation. In our study, preoperative serum IL-6 levels were positively correlated with preoperative BDI-II scores. Patients with ENS with severe depression had higher serum IL-6 levels than the rest of the population, and those with preoperative IL-6 levels > 1.985 pg/mL had a nearly 10-fold risk of experiencing severe depression. Moreover, more suicidal thoughts or attempts were noted in patients with ENS with severe depression.^11^ It may implicate that we can utilize serum IL-6 for screening patients with a probability of experiencing advanced depressive states among ENS, who may need timely and proper management to prevent unpleasant consequences.

Depression is a common psychiatric health issue affecting an estimated 5% of adults worldwide.^39^ However, approximately 70% of patients with ENS have been shown to experience depression 4,6,11 which is much higher than the global prevalence. In this present investigation, 68.6% presented a depressive status before surgical treatment. In this study we found ENS patients with a higher preoperative serum IL-6 level related to a severe status of depression and would need timely management. In the future, we should enroll depressive patients without ENS for a direct comparison of serum IL-6 levels to clarify its role in ENS or just another aspect of a biomarker for depression. Even if no significant difference detected in serum IL-6 levels between those with or without ENS in future studies, this biomarker would be still helpful clinically to screen out those in severe depressive status among ENS patients in advance for its high prevalence in this population and take timely management to prevent unpleasant consequences. Based on present findings, preoperative serum IL-6 levels did not correlate to postoperative subjective measurements, indicating that preoperative serum IL-6 level may have no role in surgical outcome prediction so far. Although no significant difference was found between pre- and postoperative serum IL-6 levels among all enrolled ENS patients, we found those in severe depression before surgical treatment showed a decrease in the serum IL-6 level with marginal significance (3.30 pg/mL vs. 1.94 pg/mL, *p* = 0.047). However, the number of this patient group was limited (only 10 received postoperative examinations). Further, the trend of postoperative change serum IL-6 and its impacts for ENS would be obtained after more patient enrollment at different timepoints after surgical treatment in future studies. Moreover, other potential neurotransmitters, such a serotonin IA receptors and neurotrophic factors, should be further investigated to search more feasible biomarkers for ENS.

Our study has some limitations. First, the sample size is relatively small. Not every patient develops ENS after turbinate reduction, and most need time to develop symptoms, which makes it difficult to increase our sample size in a short period of time. Second, due to the coronavirus disease-2019 pandemic, some patients did not complete regular postoperative follow-up or postoperative serum IL-6 measurement, making it difficult to evaluate the relationship between serum IL-6 levels and postoperative conditions, which may result in selection bias. Further studies are warranted to investigate the association between baseline serum IL-6 levels and postoperative psychiatric conditions. The efficacy of surgical treatment combined with an antidepressant regimen for patients with ENS with higher serum IL-6 levels requires further investigation.

## Conclusion

Our study showed that serum IL-6 may be a potential objective tool to screen out patients with ENS with a probability of experiencing severe psychiatric symptoms. Under these patients have more suicidal thoughts or attempts, they may require modified and timely treatment. A larger sample size and longer follow-up period are crucial for a more definite conclusion.

## Figures and Tables

**Table 1:** Demographic data, laboratory parameters, and preoperative subjective assessments

Characteristic (*N* = 35)	Mean (SD)/Number (%)
Age, years (SD)	47.0 (12.2)
Gender, *n* (male: female)	26: 9
Smoking, *n* (%)	6 (17.1)
BMI, kg/m^2^ (SD)	24.2 (3.6)
Comorbidity^a^, *n* (%)	7 (20.0)
Preoperative IL-6, pg/mL (SD)	2.5 (1.63)
Preoperative ENS6Q score, mean (SD)	17.3 (3.7)
Preoperative SNOT-25 score, mean (SD)	77.4 (17.9)
Preoperative BDI-II score	
BDI-II score, mean (SD)	23.7 (14.9)
Normal (0-13), *n* (%)	11 (31.4)
Mild (14-19), *n* (%)	5 (14.3)
Moderate (20-28), *n* (%)	6 (17.1)
Severe (29-63), *n* (%)	13 (37.1)
Preoperative BAI score	
BAI score, mean (SD)	25.5 (14.1)
Normal (0-7), *n* (%)	3 (8.6)
Mild (8-15), *n* (%)	8 (22.9)
Moderate (16-25), *n* (%)	7 (20.0)
Severe (26-63), *n* (%)	17 (48.6)

All values are reported as mean (SD) for continuous variables or number (percentage) for categorical variables, as indicated. BAI: Beck anxiety inventory; BDI-II: Beck de-pression inventory-II; BMI: Body Mass Index; ENS6Q: Empty Nose Syndrome 6-Item Questionnaire; IL-6: Interleukin-6; SD: standard deviation; SNOT-25: Sino-Nasal Outcome Test-25.^a^ Comorbidity: one with acute hepatitis, one with psoriasis, one with hyperlipidemia, one with hypertension, one with coronary artery disease, one with glaucoma, and one with pertussis.

**Table 2:** Correlation between preoperative IL-6 level and other parameters

Variables	B	95% CI	*p*-value
Age	0.37	0.06-0.94	0.028*
Smoking	-	-	0.881
BMI	-0.004	-0.16-0.16	0.981
Comorbidity	-	-	0.856
PreOP SNOT-25	0.39	0.01-0.07	0.021*
PreOP ENS6Q	0.36	0.01-0.31	0.034*
PreOP BDI-II	0.39	0.01-0.08	0.022*
PreOP BAI	0.37	0.004-0.08	0.031*

Continuous variables with simple linear regression analysis and categorical variable with Mann-Whitney *U* test. BAI: Beck anxiety inventory; BDI-II: Beck depression inventory‐II; BMI: Body Mass Index; ENS6Q: Empty Nose Syndrome 6‐Item Questionnaire; IL-6: Interleukin-6; PreOP: preoperative; SNOT-25: Sino‐Nasal Outcome Test‐25.* *p* < 0.05 statistically significant.

**Table 3:** Performance characteristics of preoperative serum IL-6 level in predicting depression status

	≤ 1.985 pg/mL (*n* = 21)	> 1.985 pg/mL (*n* = 14)	
**Severe depression (*n* = 13)**	4	9	Sensitivity = 69.2%
**Without severe depression (*n* = 22)**	17	5	Specificity = 77.3%
	NPV = 81.0%	PPV = 64.3%	

IL-6: interleukin-6; NPV: negative predictive value; PPV: positive predictive value.

**Table 4:** Logistic regression analysis for preoperative severe depression status

	Univariate analysis			Multivariate analysis			
	OR	95% CI	*p*-value		OR	95% CI	*p*-value
Age	1.02	0.97-1.09	0.430		0.98	0.91-1.06	0.598
Smoking	0.82	0.13-5.23	0.832		0.72	0.06-8.26	0.795
BMI	1.10	0.90-1.34	0.35		1.06	0.83-1.36	0.630
Comorbidity	2.82	0.52-15.32	0.231		3.54	0.52-24.16	0.197
IL-6 > 1.985 pg/mL	7.65	1.64-35.80	0.010*		9.76	1.43-66.67	0.020*
							

BMI: Body Mass Index; CI: confidence interval; IL-6: Interleukin-6; OR: odds ratio.* p < 0.05 statistically significant.
